# Mitochondrial Dysfunction in Atrial Fibrillation: The Need for a Strong Pharmacological Approach

**DOI:** 10.3390/biomedicines12122720

**Published:** 2024-11-27

**Authors:** Alfredo Mauriello, Adriana Correra, Riccardo Molinari, Gerardo Elia Del Vecchio, Viviana Tessitore, Antonello D’Andrea, Vincenzo Russo

**Affiliations:** 1 Cardiology Unit, Department of Medical and Translational Sciences, University of Campania “Luigi Vanvitelli”, Monaldi Hospital, 80131 Naples, Italy; alfredo.mauriello93@libero.it (A.M.); riccmolinari@gmail.com (R.M.); delvecchiogerardo@gmail.com (G.E.D.V.); viviana.tessitore.vt@gmail.com (V.T.); 2 Cardiology and Intensive Care Unit, Department of Cardiology, Umberto I Hospital, 84014 Nocera Inferiore, Italy; antonellodandrea@libero.it; 3 Intensive Cardiac Care Unit, San Giuseppe Moscati Hospital, ASL Caserta, 81031 Aversa, Italy; adrianacorrera@gmail.com

**Keywords:** atrial fibrillation, mitochondrial dysfunction, arrhythmia, oxidative stress

## Abstract

Despite great progress in treating atrial fibrillation (AF), especially with the development of increasingly effective invasive techniques for AF ablation, many unanswered questions remain regarding the pathogenic mechanism of the arrhythmia and its prevention methods. The development of AF is based on anatomical and functional alterations in the cardiomyocyte resulting from altered ionic fluxes and cardiomyocyte electrophysiology. Electric instability and electrical remodeling underlying the arrhythmia may result from oxidative stress, also caused by possible mitochondrial dysfunction. The role of mitochondrial dysfunction in the pathogenesis of AF is not yet fully elucidated; however, the reduction in AF burden after therapeutic interventions that improve mitochondrial fitness tends to support this concept. This selected review aims to summarize the mechanisms of mitochondrial dysfunction related to AF and the current pharmacological treatment options that target mitochondria to prevent or improve the outcome of AF.

## 1. Introduction

Mitochondria have a critical role in cellular homeostasis. They represent the “fuel” of the cell, and so, in case of mitochondrial dysfunction, there would be significant and severe alterations. In addition to specific mitochondrial syndromes, mitochondrial alterations can determine the onset of diseases with multifactorial pathogenesis. Interestingly, mitochondria could also contribute to the development of arrythmias. This selected review aims to verify whether there is a connection between mitochondrial dysfunction and the development of atrial fibrillation (AF) and whether mitochondria can become a therapeutic target of AF.

## 2. Role of the Mitochondria in Systemic Disease

The mitochondria are essential organelles in nucleated cells, involved in adenosine triphosphate (ATP) production. The oxidative phosphorylation system includes five protein complexes and two factors (coenzyme Q10 and cytochrome C) that produce ATP through electron transfer along the inner membrane of mitochondria. As a byproduct of phosphorylation, mitochondria also represent the main source of reactive oxygen species (ROS), which in great amounts could lead to mitochondrial and cell dysfunction. Mitochondrial disease can be linked to cardiovascular risk factors or include complex and massive genetic disorders caused by dysfunction of mitochondria [1]. Several cardiovascular risk factors, such as hyperglycemia, can cause augmented ROS (Table 1). In addition, mitochondrial deoxyribonucleic acid (DNA) covers only a small number of mitochondrial proteins; about 1500 proteins essential to the respiratory chain are encoded by nuclear DNA [2]. In addition to these general mechanisms of AF pathogenesis, there are studies demonstrating a primary role of mitochondrial DNA dysfunction in the pathogenesis of AF, and they are inherited according to the Mendelian model (including autosomal recessive, dominant, and X-linked patterns, with the autosomal recessive being the most common modality of transmission) [2]. The genetic basis of mitochondriopathies is extremely varied since a single-gene mutation causes some diseases, and others are secondary to multiple-gene mutations, such as in Leigh syndrome, in which 75 genes can be involved [3]. Moreover, concerning mitochondrial DNA, another source of genetic heterogeneity is caused by heteroplasmy. Heteroplasmy refers to a combination of mutated and wild-type mitochondrial DNA molecules inside human cells. The pathological phenotype can occur only in the case of more than 60% mutated DNA molecules (with a kind of threshold effect) [1,2]. In addition to these general mechanisms of AF pathogenesis, there are studies demonstrating a primary role of mitochondrial DNA dysfunction in the pathogenesis of AF. Yamazoe et al. aimed to evaluate the level of cell-free DNA (cfDNA) in AF patients and AF-mimicking models and to clarify its impact on inflammation. Nuclear and mitochondrial DNA were extracted separately and fragmented to mimic nuclear cfDNA (n-cfDNA) and mitochondrial cfDNA (mt-cfDNA). The AF group showed a higher cfDNA concentration than the non-AF group (*p* < 0.001). The copy numbers of n-cfDNA and mt-cfDNA were higher in the AF groups than in the non-AF groups; the difference particularly of mt-cfDNA was evident (*p* = 0.011 and *p* < 0.001, respectively). Administration of total cfDNA and mt-cfDNA to macrophages significantly promoted the expression of IL-1β and IL-6 via TLR9, whereas n-cfDNA did not. The induction of cytokine expression by methylated mt-cfDNA was lower than that by unmethylated mt-cfDNA. Collectively, AF was associated with an increased level of cfDNA, particularly mt-cfDNA. Poorly methylated mt-cfDNA released from cardiomyocytes may be involved in the sterile systemic inflammation accompanied by AF [3].

## 3. Mitochondrial Diseases Correlated with Atrial Fibrillation

Mitochondrial diseases often represent a clinical challenge because of the extreme phenotypic variability. Mitochondrial dysfunction affects all those tissues with high energy requests (skeletal muscle, heart, nervous system, eyes, and other sensory tissues), and they encompass a wide spectrum of clinical manifestations. Cardiac involvement is a common clinical finding in mitochondrial diseases and can often influence the prognosis in a significant way. The main cardiac manifestations include hypertrophic, dilated cardiomyopathy (often present together), and arrhythmic abnormalities such as AF. This phenotype is also in the different mitochondriopathies [7]. Some of the most common mitochondriopathies correlated with AF that arise in childhood are Leigh syndrome (OMIM: #256000) and Sengers syndrome (OMIM: #212350). They are characterized by non-specifical signs and symptoms such as fatigue, hypotonia, gastrointestinal discomfort, failure to thrive, and neurological manifestations, with which many other specifical clinical manifestations are associated. In adulthood, the most common forms are Kearns–Sayre syndrome (OMIM: #530000), MELAS syndrome (OMIM: #540000), and Leber hereditary optic neuropathy (OMIM: #535000). There is also, in cases of late onset, a wide spectrum of clinical involvement. MELAS syndrome, for example, typically develops around the age of forty or later in adulthood [1,2,7,8,9,10]. In the Table 2 are summarized the main mitochondrial diseases with cardiac involvement.

## 4. Acquired Mitochondrial Dysfunction

**Figure 1:**
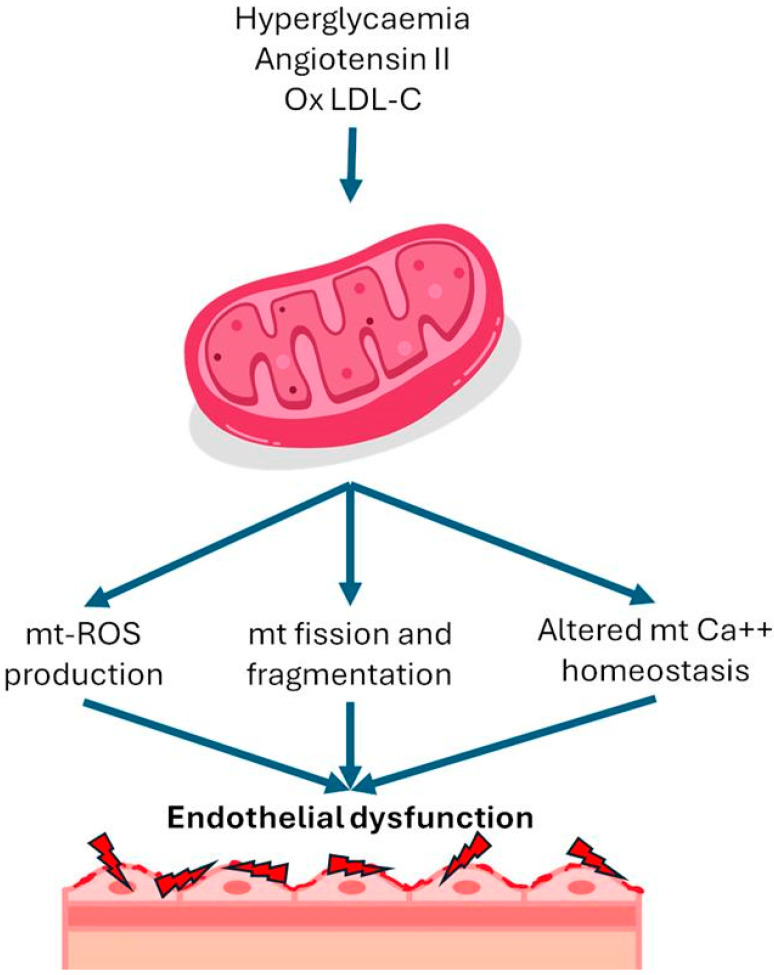
Mitochondrial alterations in endothelial dysfunction. LDL-C, lipoprotein low density-concentration; Mt, mitochondrial; ROS, reactive oxygen species.

In addition to genetic disorders of the mitochondria, several cardiovascular risk factors can cause augmented ROS production in endothelial cells. The main proven risk factors are hyperglycemia, increased angiotensin II, and oxidized LDL. Hyperglycemia has been shown to trigger an overproduction of mitochondrial ROS [11]. Under normal circumstances, protons are shuttled into the intermembrane space of mitochondria during the electron transport chain, establishing a proton gradient that fuels ATP synthesis. However, in high intracellular glucose levels, additional electron donors like nicotinamide adenine dinucleotide (NADH) and flavin adenine dinucleotide hydride (FADH)-2 enter the electron transport chain, boosting oxidation in the tricarboxylic acid cycle. This culminates in an increased voltage gradient across the mitochondrial membrane until it reaches a critical threshold. When this threshold is surpassed, electron transfer within complex III of the electron transport chain is blocked, causing electrons to revert to coenzyme Q and subsequently donate electrons to oxygen molecules, thereby generating superoxide [4]. Angiotensin II induces mitochondrial dysfunction through a protein kinase C-dependent pathway by activating endothelial nicotinamide adenine dinucleotide phosphate (NADPH) oxidase 2 (NOX2). In this process, mitochondrial protein kinase C (PKC) epsilon is a downstream target of NOX2, and it activates mitochondrial ATP-sensitive potassium channels, leading to mitochondrial reverse electron transfer and subsequent generation of superoxide [12]. In addition to the factors mentioned above, several other cardiovascular risk factors, including oxidized low-density lipoprotein (LDL) and turbulent flow, have been associated with an increase in mitochondrial ROS levels in endothelial cells [6] (Figure 1).

## 5. Cellular Alterations in the Heart and Electrogenesis of Atrial Fibrillation

The development of an arrhythmia is related to several mechanisms, i.e., augmented or reduced automaticity, triggered activity, and re-entrant circuit [13]. The underlying mechanisms that initiate and perpetuate AF are not fully elucidated. However, abnormalities in calcium ion (Ca^2+^) flux and oxidative stress are thought to play a central role in the pathophysiology of AF. Mitochondria are the major producers of cellular adenosine triphosphate (ATP) in cardiac myocytes, and both Ca^2+^ and adenosine diphosphate (ADP) are key regulators of respiratory flow to adapt energy supply to the ever-changing demands in the heart [14].

### ATP Homeostasis, ROS Production, and Atrial Fibrillation

**Figure 2:**
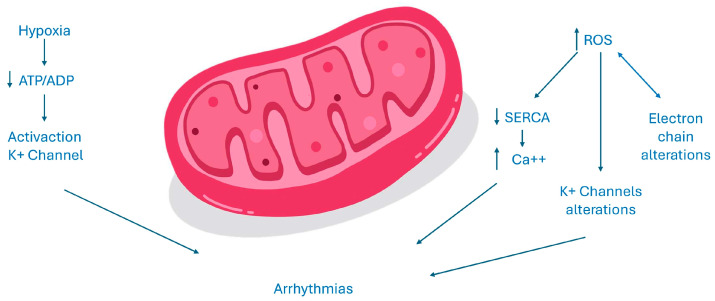
Electrogenesis of AF in mitochondrial dysfunction. ADP: adenosine diphosphate; ATP: adenosine triphosphate; ROS: radical oxidative species; SERCA: Sarco-Endoplasmic Reticulum Calcium ATPase; ↑: increase; ↓: reduction.

The dysfunction of mitochondria has a negative impact since it could reduce ATP production and increase ROS production. The reduction in ATP source alters all enzymes and ion transporters that are ATP-dependent and thus the normal cell excitability. An increased ADP/ATP ratio as an acidosis status or a variation of magnesium concentration, all conditions present in the case of myocardium ischemia, could activate sarcKATP channels. The inward potassium current causes a reduced action potential duration and could be cytoprotective since it reduces calcium overload. However, this could be detrimental too, increasing the risk of arrhythmias since an open status of these channels shortens the duration of action potential and the duration of effective refractory period and creates an electrical dispersion, which increases the risk of re-entrant arrhythmias. Moreover, the increase in ROS could activate a maladaptive response in the long term and could alter normal gene expression, inducing cell death [13]. ROS could increase the release of calcium interfering with ryanodine receptor 2 (RyR2), thus inhibiting the activity of Sarco-Endoplasmic Reticulum Calcium ATPase (SERCA). SERCA is the main channel involved in the diastolic relaxation of the myocardium, absorbing calcium into the reticulum. Moreover, ROS induces a kind of vicious circle in which the damage of all the components of the transfer electron chain and ion channels leads to an increased production of ROS, too. The final effect is an intracellular calcium overload that represents a proarrhythmic stimulus [15]. Furthermore, oxidative stress increases arrhythmic burden, activating the late sodium current, which thereafter leads to early depolarizations, prolonging the duration of action potential. ROS could also interfere with potassium balance in different ways. They could modify gene expression of potassium currents but also could modify potassium channels functioning, acting on kinase or phosphatase proteins, which regulate the phosphorylation status of these channels. The cell integrity and the normal function of the myocardial syncytium are essential, especially in leading to the transmission of cardiac impulses. The linkage between cardiomyocytes is regulated by connexins, which are present in three different isoforms in myocardial tissue. An increased stimulus to ROS production that may be secondary to different pathological scenarios associated with the activation of the RAS system causes downregulation of connexin 43. The dysregulation of gap junctions could impair electrical conduction, increasing arrhythmic events through an increased electrical dispersion [13,15]. Recent publications have explored the correlation between vascular function and AF, suggesting the involvement of endothelial dysfunction in AF development [15,16,17,18]. Endothelial dysfunction has been demonstrated to contribute to the initiation and perpetuation of the atrial arrhythmic substrate regulating immune cell infiltration and inflammation within the cardiac tissue, augmenting the fibrous burden of the atria [19,20] and promoting oxidative stress through the overproduction of reactive oxygen species, with known arrhythmogenic effects [19]. Figure 2 summarizes electrogenenis of AF in mitochondrial dysfunction.

## 6. Drugs with Mitochondrial Effects

Current medications and nutraceutical products utilized in cardiovascular medicine target these issues, with existing in vitro and animal studies indicating positive impacts on mitochondrial function and cellular metabolism. Nevertheless, to broaden their clinical applications in this setting, rigorous randomized, double-blind, placebo-controlled trials specifically within the context of AF are imperative for medications that influence mitochondrial function. These trials are essential to validate and establish the efficacy of such interventions, paving the way for wider adoption in clinical practice [21]. The therapeutic strategies of influence on mitochondrial function and AF are shown in Table 3.

### 6.1. Oral Hypoglycaemic Agents

Oxidative stress and mitochondrial dysfunction due to chronic hyperglycemia are a crucial element in the pathogenesis and progression of diabetes and its complications [22]. However, it is not by chance that many studies about the effects of oral hypoglycemic agents on oxidative damage have been conducted.

SGLT2 inhibitors, targeting the sodium-glucose co-transporter 2, enhance glycemic control and induce natriuresis and uricosuria. Beyond diabetes management, these inhibitors demonstrate favorable cardiovascular outcomes, especially in heart failure (HF) and chronic kidney disease (CKD). Subgroup analyses indicate benefits in preventing arrhythmias, including AF, suggesting potential pleiotropic effects in cellular metabolism and mitochondrial function. Li et al. demonstrated that in diabetic mice, the use of empagliflozin suppressed oxidative stress and myocardial fibrosis by inhibiting the transforming growth factor-beta (TGF-beta/Smad) pathway and activating the nuclear factor erythroid 2-related factor 2/antioxidant responsive element (Nrf2/ARE) signaling [23]. In Shao et al.’s study, SGLT2 inhibition with empagliflozin restores mitochondrial membrane potential, enhances the respiratory rate, and promotes mitochondrial biogenesis through increased expression of peroxisome proliferator-activated receptor-gamma coactivator 1 (PGC-1), nuclear respiratory factor-1 (NRF-1), and mitofusin-1 (Mfn-1). This improvement correlates with reduced reactive oxidative species (ROS) synthesis, systemic inflammation, atrial fibrosis, and cardiomyocyte hypertrophy, resulting in a significant 36.8% reduction in tachypacing-induced AF susceptibility [24]. Analog results were found by Koizumi et al. showing that the treatment lead to a reduction in inflammation, atrial fibrosis, atrial tachyarrhythmia inducibility, and atrial tachyarrhythmia duration and normalized the interatrial conduction time in parallel with a decrease in mitochondrial ROS generation, increase in superoxide dismutase (SOD) activity, and increase in mitochondrial content through adenosine monophosphate-activated protein kinase (AMPK) signaling pathways [25]. Recently, Zhao et al. analyzed the effects of dapagliflozin in rats with a sepsis-like condition and demonstrated significant improvement in myocardial injury and susceptibility to AF correlated with the activation of the Nrf2 signaling pathway, leading to a reduction in oxidative stress [26].

Glucagon-like peptide-1 (GLP1)-RA acts as a similar of the hormone GLP1, which stimulates glucose-dependent insulin secretion. Nuamnaichati et al. demonstrated that GLP-1 receptor activation has antioxidant and antiapoptotic effects, reducing intracellular and mitochondrial ROS production in cardiomyoblasts. This improvement is mediated through the phosphoinositide 3-kinases/protein kinase B (PI3K/PKB) signaling pathway [28]. In addition, GLP1-ameliorated interleukin-1 beta (IL-1β)-induced ROS production reduced nicotinamide adenine dinucleotide phosphate oxidase-4 (NOX-4) expression and increased the expression of SOD-1 and glutathione peroxidase [27,29]. In diabetic animal models, the amelioration of mitochondrial function due to GLP1-RA administration led to a significant reduction in collagen deposition, improving cardiac remodeling, diastolic function and the incidence of AF [29] However, a meta-analysis showed no significant reduction in the risk of major arrhythmias in more than 60,000 diabetic patients treated with GLP1-RA, including AF (relative risk (RR) = 0.96, 95% confidential interval (CI) (0.86, 1.07), *p* = 0.43) [27], and a French cohort study showed an increased risk of AF in patients treated with GLP1-RA [32]

The dipeptidyl peptidase-4 (DPP-4) inhibitors belong to a novel class of oral antidiabetic medications that target the incretin system. DPP-4 is an enzyme responsible for deactivating gastric inhibitory polypeptide (GIP) and GLP-1. The nationwide cohort study taking place in Taiwan and including over 90,000 diabetic patients already treated with metformin showed that the use of DDP4i as a second-line drug (mostly sitagliptin) lowered the risk of new-onset AF compared with non-DPP4i users (hazard ratio (HR): 0.65; 95% confidential interval (CI) 0.56–0.76; *p* < 0.0001) [33]. In vitro studies demonstrated that the expression of DDP-4 is augmented in cells exposed to hypoxic conditions, leading to the generation of ROS and inner mitochondrial membrane potential reduction. Therefore, inhibition of this enzyme could have cardioprotective effects mostly due to the attenuation of oxidative stress and amelioration of mitochondrial function [33]. Zhang et al. found that in rabbits with alloxan-induced diabetes mellitus (DM), the DDP-4 inhibitor alogliptin demonstrated significant protective effects on mitochondria, reducing the production rate of mitochondrial reactive oxygen species, preventing membrane depolarization, and alleviating mitochondrial swelling. Additionally, alogliptin improved mitochondrial biogenesis through the adiponectin/AMP-activated protein kinase pathway, specifically activating PGC-1/NRF1 signaling. These positive mitochondrial modifications contributed to a reduction in AF inducibility [34]. Positive effects were observed also for linagliptin in a canine AF model in which the treatment determined a suppression of the inducibility of AF and atrial fibrosis alongside a suppression of the ROS expression [35]. However, in the recent meta-analysis conducted by Patoulias et al. involving 52,520 patients from six trials comparing DPP-4i with a placebo, the treatment did not seem to confer any significant cardiovascular benefits and did not affect the risk for AF (RR = 0.95, 95% CI: 0.78–1.17, I = 0%), while it was associated with a significant increase in the risk for atrial flutter, equal to 52% (RR = 1.52, 95% CI: 1.03–2.24, I2 = 0%) [36].

In 2022, Chan et al. published a meta-analysis including 2,826,059 patients in which SGLT2i treatment was associated with a lower risk of new-onset AF in participants with type 2 DM compared with either DPP4i (hazard ratio (HR):0.90; 95% CI 0.84–0.96; *p* = 0.0028) or GLP-1RA (HR 0.74; 95% CI 0.63–0.88; *p* = 0.0007), with no statistically significant difference between GLP-1RA and DDP4i (HR 1.01; 95% CI 0.86–1.19; *p* = 0.8980) [37].

Metformin primarily exerts its metabolic effects in the liver, where it reduces glucose and lipid synthesis through the phosphorylation and activation of 5′ AMP-activated protein kinase (AMPK). Furthermore, AMPK activation leads to glucose transporter-4 mediated glucose uptake, contributing to higher systemic insulin sensitivity. The impact of metformin on atrial remodeling has been investigated, as evidenced by a study analyzing data from 645,710 patients with DM2 over a 13-year follow-up period from the Taiwan National Health Insurance Research Database. The findings revealed a 19% decrease in the incidence of AF with metformin use (HR 0.81, 95% CI 0.76–0.86, *p* < 0.001)) [39]. Experimental studies involving pacing-induced AF offer insights into the possible molecular mechanism of this evidence. Metformin activated AMPK and Src kinase and normalized connexin expression, reducing pacing-induced AF effects and preventing atrial remodeling by activating the AMPK/PGC-1/peroxisome proliferator-activated receptor (PPAR)-pathway. Metformin’s electronegativity concentrates it in the mitochondria, influencing cellular energy metabolism and preserving mitochondrial function, with improved oxygen consumption and augmented activity of complexes I, II, and IV and upregulation of PGC-1alfa [19,40].

Thiazolidinediones (TZDs) constitute a medication group in DM treatment that impacts mitochondrial function by acting as PPAR-agonists to reduce insulin resistance. Studies about their effect on AF have shown conflicting results. Clinical evidence from Danish nationwide registries published by Pallisgaard et al., with over 100,000 diabetic patients, revealed that TZDs reduced AF incidence by 24%, when adjusted for age, sex, and comorbidities, compared to other second-line antidiabetic treatments (HR 0.76, 95% CI 0.57–1.00, *p* = 0.047) [41]. A meta-analysis combining three randomized clinical trials and four observational studies supported the preventive potential of TZDs against AF, showing an overall reduction of 30% (odds ratio (OR): 0.73, 95% CI 0.62–0.87, *p* = 0.0003), with a 23% reduction in new-onset AF and a 59% risk reduction in AF recurrence [41]. Animal models such as alloxan-induced DM in rabbits demonstrated that TZDs attenuated atrial remodeling, reduced AF inducibility, and improved ion channel function (ICa and INa) [42]. Xu et al. found that pioglitazone pretreatment decreased AF duration and age-related atrial remodeling, suggesting protective mechanisms involving the upregulation of antioxidant pathways and inhibition of mitochondrial apoptotic signaling [56]. However, in patients with coronary artery disease undergoing TZD or other second-line medications during a median follow-up of 4.2 years, TZDs did not affect AF prevalence [43]. Moreover, in a small, randomized, prospective study, TZDs did not affect AF recurrence after electrical cardioversion [44].

### 6.2. Hypolipidemic Drugs

Statins act as 3-hydroxy-3-methylglutaryl-coenzyme A reductase inhibitors and are the cornerstone of treatment in dyslipidemias. Beyond the effect on lipidic metabolism, statins also exert a pleiotropic cardioprotective effect. Statins can offer protection against AF not only by diminishing the burden of vascular disease but also by addressing atrial remodeling thanks to anti-inflammatory, anti-oxidative, anti-proliferative, and antithrombotic actions. Additionally, statins may enhance endothelial function and neurohormonal regulation [57]. According to the Taiwan National Health Insurance research database, statins demonstrated a 13% decrease in the risk of new-onset AF (HR: 0.935; 95% CI: 0.877–0.998; *p* = 0.0427) [45]. However, a Spanish registry study by Cabratosa-Alves et al. reported minimal protective effects of statins against new-onset AF in cases of lone hypertension without vascular ischemic disease [46]. A meta-analysis conducted by Fang et al. revealed an overall significant reduction in the risk of AF incidence/recurrence (odds ratio (OR) = 0.49, 95% CI 0.37–0.65; *p* < 0.00001); this effect appeared to be more pronounced in secondary prevention (OR = 0.34, 95% CI 0.18–0.64; *p* < 0.0001) than in primary prevention (OR = 0.54, 95% CI 0.40–0.74; *p* < 0.0001) [47]. Inflammation and abnormal oxidative stress were identified as key pathophysiological features linked to atrial remodeling and heightened myocardial tissue inflammation, leading to AF onset, recurrence, and persistence [57]. The mechanisms by which statins exert their antioxidant action are suppression of the activity of rhodopsin/Rho-associated protein kinase (Rho/ROCK) pathways, activation of PI(3)K/PKB pathway and inhibition of GTPase Ras-related C3 botulinum toxin substrate 1 (Rac1) required for nicotinamide adenine dinucleotide phosphate oxidase (NADPH oxidase) activity as well as of the expression of mRNA expression of NADPH oxidase subunits (Nox1, p22phox) and indirectly by reduction in pro-inflammatory cytokines production [57].

Recent evidence suggests a potential association between proprotein convertase subtilisin/Kexin type 9 (PCSK9) and oxidative stress, particularly about oxidized LDL-induced endothelial cell apoptosis and the regulation of PCSK9 expression by ROS. Furthermore, mitochondrial ROS appear to influence the interaction between lectin-type oxidized LDL receptor 1 (LOX-1) and PCSK9, which are key players in atherosclerosis. Evolocumab, a PCSK9 inhibitor, demonstrated a significant reduction in oxidative stress-related cytotoxicity and atherosclerotic progression in both cellular and animal models. Safeian et al. found that Evolocumab led to a significant reduction in cytotoxicity induced by H_2_O_2_ in human umbilical vein endothelial cells [58]. In a mouse model, Evolocumab lowered atherosclerotic progression, reducing oxidative stress through promoting macrophage autophagy [59]. It was proposed that the antioxidant activity of PCSK9i may be at least in part mediated by NAD-dependent deacetylase sirtuin-3 (SIRT3), which counteracts mitochondrial ROS accumulation [60]. A significant reduction in leucocyte ROS production (H_2_O_2_) was observed in a sample of 18 hypercholesterolemic patients after 2 weeks of treatment with Evolocumab (*p*-value = 0.004) [61]. However, the antioxidant effects of PCSK9 inhibitors have not yet been investigated in the context of AF.

Fibrates, acting as PPAR-alfa agonists, are commonly utilized to address hypertriglyceridemia, decrease hepatic apolipoprotein C-III (apoC-III) levels, and enhance lipoprotein lipase-mediated lipolysis. By operating on the PPAR/PGC-1 pathway, these medications influence mitochondrial function. In experimental AF models, fenofibrate contributes to mitigating metabolic remodeling by regulating the PPAR-/sirtuin route 1/PGC-1, effectively reversing the shortening of the atrial refractory period [48].

Omega-3 fatty acids are used in the treatment of hypertriglyceridemia and exhibit potent anti-inflammatory effects by replacing arachidonic acid in cell membranes, especially eicosapentaenoic acid and docosahexaenoic acid. The direct impact of omega-3 fatty acids on ionic channels, coupled with their modulation of the cell membrane properties, could influence the occurrence of AF. Supplementation with these fatty acids affects ion channel function, influencing cardiac action potentials, stabilizing electrical activity, and prolonging the refractory period of cardiomyocytes thanks to the ability to reduce oxidative stress in heart cells and to preserve cell integrity. However, the efficacy of omega-3 fatty acids in preventing AF may vary based on individual conditions and clinical backgrounds [48].

### 6.3. Others

Trimetazidine (TMZ), an approved anti-anginal drug for treating ischemic heart disease, exerts beneficial effects by enhancing cellular energetic balance. It achieves this by inhibiting long-chain 3-ketoacyl coenzyme A thiols, a key player in mitochondrial fatty acid oxidation, redirecting mitochondrial substrate utilization toward glucose and thereby improving ATP synthesis. However, it seems to influence many aspects of mitochondrial function [21]. In ischemic conditions, trimetazidine (TMZ) directly activates complex I in the respiratory chain, enhancing electron transport chain (ETC) function with a significant reduction in the production of ROS; additionally, in ischemic conditions, TMZ normalizes the expression of factors regulating mitochondrial biogenesis, such as PPAR and PGC-1α, and adjusts the expression of Mfn-1, dystrophin-related protein-1 (Drp1), and mitochondrial dynamin-like GTPase (Opa-1), impacting mitochondrial fusion/fission dynamics [49]. In addition, preclinical studies have suggested an antiarrhythmic activity. Using a dog model of HF, Li et al. suggested that TMZ may prevent tachycardia-induced atrial ultrastructural remodeling, decrease AF inducibility, and shorten AF duration [50]. However, it remains unclear if TMZ’s protective effects in non-ischemic conditions are related to improvements in mitochondrial function [21].

Ranolazine functions mainly as a late sodium channel influx inhibitor during repolarization, leading to decreased intracellular sodium and calcium concentrations and thus to reduced oxygen consumption. Additionally, ranolazine is thought to act as a partial fatty acid oxidation inhibitor, contributing to the attenuation of oxidative stress. By the action on sodium and potassium channels, ranolazine showed antiarrhythmic proprieties, particularly at the atrial level. In a meta-analysis, the combination of ranolazine with amiodarone significantly increased the sinus rhythm restoration rate compared to amiodarone alone (RR = 2.87, 95% CI 2.48–3.32, *p* < 0.05) in patients with LV systolic dysfunction [51]. Ranolazine’s suggested mechanism of action involves extending post-repolarization refractoriness and slowing conduction velocity. Beyond these electrophysiological effects, studies have demonstrated that ranolazine enhances mitochondrial function, mitigates oxidative stress, and inhibits apoptosis [21]

Carvedilol exhibits a mild β1-blocking selectivity that transitions to non-selective at elevated doses. Additionally, it possesses α1-blocking and antioxidant attributes, influencing diverse ion channels and currents. Notably, it outperforms selective β1 blockers like metoprolol and atenolol in the suppression of post-operative AF. The proposition that carvedilol excels over other beta-blockers in AF treatment is partially elucidated by its antioxidative effects [48].

In the atrial tissues of individuals, AF-increased levels of angiotensin II (Ang II), Ang II receptor, aldosterone, and an augmented activity of angiotensin-converting enzyme (ACE) were found [57]. As already mentioned in Section 4, the expression of ATII could lead to ROS production. Therefore, inhibiting Ang II production aids in reducing oxidative stress (OS) in vascular structures [48]. Moreover, the ATII-induced production of H_2_O_2_ triggers the binding action of nuclear factor kappa B (NF-κB) to the sodium channel protein type 5 subunit alpha (SCN5A), reducing the inward current of sodium (INa). The downregulation of SCN5A and INa alters the cellular electrical propriety, promoting arrhythmias. This can be prevented by renin–angiotensin system (RAS) inhibition with angiotensin-converting enzyme inhibitors (ACE-I) or angiotensin II receptor type 1 (AT1R) blocker [57]. Recently, Zhao et al. highlighted the favorable effects of aliskiren (ALS) in attenuating atrial remodeling and diminishing susceptibility to AF by reducing inflammation and oxidative stress via PI3K/PKB in dogs subjected to tachycardic pacing [62].

Xanthine oxidase (XO) plays an instrumental role in redox signaling across various cardiovascular disorders, particularly emphasizing its involvement in AF. The effects of drugs that act on XO were thought to be beneficial [48]. In their investigation, Xu et al. explored the impact of febuxostat and allopurinol, i.e., XO inhibitors, on AF susceptibility. Both febuxostat and allopurinol demonstrated significant suppression of atrial remodeling associated with hypertension and the perpetuation of AF in a murine model. This potential effect was attributed to the inhibition of the ox-Ca^2+^-calmodulin-dependent protein-kinase type-II (CaMKII) signaling pathway, a regulator of heart contraction. Moreover, febuxostat exhibited antioxidant effects by directly combating reactive oxygen species (ROS) [52].

Inflammation and oxidative stress are strictly related. Recent clinical trials have revealed that corticosteroids and colchicine may prevent AF development and support the fundamental impact of inflammatory pathways in managing AF [30,63]. A meta-analysis by Liu et al., including fourteen studies with 13,803 patients, showed that corticosteroids significantly decreased the risk of post-operative AF (relative risk (RR), 0.7; 95% CI 0.55–0.89; *p* = 0.003) [31]. In a murine model, colchicine taken up by leucocytes inhibits cytokine and interleukin expression and modulates leucocyte superoxide production [63]. Table 3 summarizes the mentioned drugs [21,30,31,38,48,53,54,55,64,65,66,67].

## 7. Nutraceutical with Mitochondrial Effects

Ubiquinone, i.e., coenzyme Q10 (CoQ10), is a coenzyme crucial for mitochondrial complexes engaged in ATP production. CoQ10 stands out as one of the most widely used nutritional supplements. Its significance in cellular bioenergetics has been extensively researched in both animal and human studies. The proposed application of CoQ10 in patients with cardiovascular disease primarily focuses on two clinical entities: statin-associated muscle symptoms (SAMS) and HF [54,64]. However, emerging evidence hints at its potential in AF prevention. In HF patients, Zhao et al. demonstrated that CoQ10 treatment significantly reduced AF incidence compared to placebo over 12 months (6.3% vs. 22.2%; *p* = 0.02) [55]. While a small, randomized, controlled trial suggested a potential reduction in post-operative AF with short-term CoQ10 treatment, these findings lacked consistent support in subsequent studies, as outlined in a meta-analysis. However, given its good tolerability and favorable safety profile, CoQ10 could be considered as an adjunctive therapy to mitigate AF risk in specific scenarios [21].

Treatment with vitamin C has been shown to decrease the incidence of post-surgical AF as well as arrhythmia recurrence after electrical cardioversion in a prospective study involving 44 patients (*p* = 0.024). N-acetyl cysteine was shown to reduce the risk of AF in dogs undergoing tachycardic pacing by increasing the density of L-type calcium current [65].

L-glutamine is increasingly recognized as a potential nutraceutical for AF treatment thanks to its ability to reduce ROS production and stabilize the microtubule network through heightened heat-shock protein (HSP) expression [66]. Costunolide, a sesquiterpene lactone renowned for its anti-inflammatory and anti-fibrotic properties, effectively mitigates inflammation and fibrosis induced by Ang II in mice. Notably, costunolide has demonstrated the ability to preserve mitochondrial function and reduce oxidative stress [48]. Andrographolide, an active molecule in the plant *Andrographis paniculata*, has numerous pharmacological properties. It reduces heart cell apoptosis, improves mitochondrial function, has antioxidant properties, and regulates inflammation and calcium flow [48]. Table 4 summarizes the mentioned nutraceutical drugs.

## 8. Experimental Drugs with Mitochondrial Effects

Elamipretide, also known as Bendavia, MTP-131, or SS-31, is a pioneering mitochondria-targeted drug under clinical investigation for primary mitochondrial myopathy [67,68] and in HF treatment. It enhances mitochondrial energetics and reduces reactive oxygen species, potentially by stabilizing the mitochondrial membrane and cytochrome C [69]. Despite initial positive studies in animals and humans with HF showing potential benefits, a recent phase 2 clinical trial with repeated elamipretide administration over 28 days in a small cohort of heart failure patients did not demonstrate improvement in left-ventricular ejection fraction [70]. KL1333, by elevating NAD+ levels and activating AMPK/PGC-1 signaling, enhances mitochondrial function and reduces oxidative stress in fibroblasts derived from patients with mitochondrial encephalomyopathy, lactic acidosis, and stroke-like episodes. Conversely, the medication KH176 demonstrates efficacy in lowering cellular ROS levels. This protective mechanism involves interaction with the thioredoxin system/peroxiredoxin enzyme pathway. Idebenone, a type of synthetic coenzyme Q10, exhibits the potential for treating various conditions associated with impaired mitochondrial function. While it demonstrated cardioprotective effects in an animal model of ischemia/reperfusion, further exploration is necessary to understand its broader impact on heart diseases [71].

In the realm of potential therapies, gene therapy represents an experimental avenue, albeit in its early stages. Genetic constructs, typically delivered through adenoviral vectors, can be transported to the myocardium via direct intramyocardial injection, epicardial gene painting, or intracoronary infusion. These gene-based approaches tested in animal models of AF have shown success in restoring sinus rhythm or improving ventricular rate control. However, their introduction into clinical practice is still premature at this stage [21].

## 9. Effect of Anticoagulant Drugs on Mitochondrial Function

Oral anticoagulants are essential for preventing stroke and systemic embolisms in AF patients. Direct oral anticoagulants (DOACs) are now preferred over vitamin K antagonists (VKAs) due to their similar efficacy and better safety profile. Consequently, DOACs have received a class I recommendation in AF management guidelines, while VKAs are reserved for specific conditions like mechanical prosthetic valves, severe mitral stenosis, or antiphospholipid syndrome (APS) [72,73]. These guidelines are based on data from randomized clinical trials (RCTs) and real-world studies of underrepresented patient subgroups [74,75]. DOACs work by inhibiting activated factor X (e.g., rivaroxaban, edoxaban, and apixaban) or thrombin (e.g., dabigatran). Furthermore, FXa and FIIa have “pleiotropic” effects, including influence on mitochondrial function and regulation of oxidative stress, through the activation of the protease-activated receptors (PARs) family [76]. Thrombin primarily activates PAR-1 but also PAR-3 and PAR-4, while FXa activates PAR-1 and PAR-2. Activation of PAR receptors triggers pro-inflammatory and pro-fibrotic effects in various cell types, mediating conditions such as atherosclerosis, atrial remodeling, cardiac hypertrophy, and chronic inflammatory pulmonary disorders, all of which may contribute to the incidence of AF [77,78]. Mitochondrial dysfunction, oxidative stress, inflammation, and coagulation are closely intertwined. ROS operate at multiple levels in the coagulation cascade, regulating endothelial functions, platelet activation, and the production of coagulation factors. This creates a vicious cycle, elevating thrombotic risk in conditions associated with oxidative stress [79]. Consequently, interest has been piqued regarding the potential effects of antithrombotic therapies on mitochondrial function and REDOX homeostasis. Even if studies conducted specifically on AF in mitochondrial disease are lacking, both in vitro and in vivo studies have suggested that direct oral anticoagulants possess antioxidant properties acting on mitochondrial function that may contribute to their beneficial effects on clinical outcomes [75,80,81]. These effects could, at least in part, contribute to the advantageous effects of DOACs compared to VKA. Indeed, warfarin was found to cause mitochondrial damage in lymphocytes and reduce the cellular ATP levels in hepatocytes, leading to compromised viability [82].

Rivaroxaban has been extensively investigated for its potential antioxidant properties in experimental and animal models. Preclinical studies using human umbilical vein endothelial cells (HUVECs) have demonstrated a dose-dependent reduction in ROS production and other oxidative stress biomarkers with Rivaroxaban treatment [83,84,85]. Similar findings were observed in human atrial cells [86]. Preclinic studies by Zekri-Nechar et al. have demonstrated that rivaroxaban, when used in combination with cardio-aspirin, improved mitochondrial functionality in human coronary artery endothelial cells (HCAECs) exposed to high glucose. This improvement was attributed to increased mitophagy promotion, enhancement in mitochondrial membrane potential, and reduction in ROS production [87]. Animal models showed that rivaroxaban may safeguard mitochondria by modifying the expression levels of various genes linked to mitochondrial function in angiotensin II-infused KKAy mice. Furthermore, it may alleviate the angiotensin II-induced decrease in cardiac ROS levels and ATP production [5]. In rat kidney mitochondria, rivaroxaban’s effects were found to be dose-dependent: At low concentrations, it induced mitochondrial dysfunction and oxidative stress by reducing the activity of mitochondrial succinate dehydrogenase and the mitochondrial membrane potential while increasing ROS production, mitochondrial swelling, and cytochrome C release. Conversely, at high concentrations, these effects were averted [88]. In a human ex vivo study focusing on abdominal aortic aneurysmal sites with intraluminal mural thrombus, Rivaroxaban improved the enzymatic activity of citrate synthase and cytochrome C oxidase, i.e., biomarkers of mitochondrial density and respiration, respectively, in the same model [89].

Numerous studies have highlighted the pleiotropic effects of Edoxaban, encompassing not only its anti-inflammatory and antioxidant properties but also its antifibrotic and anti-remodeling effects [47,90]. Regarding the effects on mitochondria, Bukowska et al. demonstrated that in exposure to factor Xa in the human lung carcinoma cell line A549, edoxaban prevented activated clotting factor X-induced mitochondrial impairment by augmenting mitochondrial oxygen consumption during maximal oxidative phosphorylation and, consequently, mitochondrial ATP production [91]. Moreover, Edoxaban showed a beneficial impact on AF induction and duration. In a canine model of congestive HF, edoxaban treatment attenuated atrial fibrosis and reduced the duration of AF episodes induced by ventricular tachypacing (VTP). It also suppressed PAR-2 and fibronectin upregulation, indicating inhibition of AF progression and structural remodeling. Additionally, in a murine model, Edoxaban mitigated vulnerability to AF episodes induced by AngII, potentially through antioxidant mechanisms [77].

Information regarding the consequences of apixaban use on mitochondrial functionality is lacking. Limited data exist about its antioxidant effects. A preclinical in vitro study conducted by Torramade-Moix et al. shed light on this aspect. In the study, HUVECs and human dermal microvascular endothelial cells (HMECs-1) exposed to uremic plasma showed normalized ROS levels following pretreatment with apixaban [92]. Additionally, Durmaz et al. demonstrated in a sample of 35 Wistar albino rats that administration of direct oral anticoagulants, including apixaban, alongside rivaroxaban and dabigatran resulted in increased total antioxidant capacity and decreased total oxidant status [93].

Dabigatran, a direct inhibitor of thrombin, has also shown potential antioxidative activity. In a HUVECs model, both dabigatran and rivaroxaban reduced ROS levels and total oxidative stress (TOS). Additionally, dabigatran mitigated oxidative damage of pyrimidines induced by oxysterol to levels comparable to control cells [93,94,95,96,97,98,99]. This antioxidative effect could be at least in part due to improvement in mitochondrial function. However, in a study using a rat gastric epithelial cell line, dabigatran induced cytotoxic effects mediated through increased ROS generation, decreased mitochondrial membrane potential, and elevated lipid peroxidation [100]. Table 5 summarizes the mentioned DOAC drugs.

## 10. Conclusions

Mitochondropathy and cardiac arrhythmias have a close etiopathogenetic relationship. In addition to mitochondrial diseases, mitochondrial dysfunction can also cause the development of cardiac arrhythmias, including AF. Several risk factors, such as hyperglycemia and dyslipidemia, can be causes of mitochondrial disease. Several drugs and nutraceuticals act indirectly at the mitochondrial level and can represent a therapeutic approach for the treatment of cardiac arrhythmias. Our narrative review data were extrapolated from several sub-analyses, so randomized clinical trials with specific hypotheses are needed to validate these findings. Based on the data from our narrative review, metformin, iSGLT2, statins, trimetazidine, beta-blockers, ACE-I, ARBs, and AT1R antagonist appear to be the most promising drugs.

## Figures and Tables

**Table 1:** Risk Factor of Mitochondrial Disease through increasing ROS.

Risk Factors of Mitochondrial Disease
Smoke [3]
Hyperglicemia [4]
Fatty foods [3]
Sedentariety [3]
Alcohol [3]
Angiotensin II [5]
Use of drugs [3]
Dyslipidemia [6]

ROS: radical oxidative species.

**Table 2:** Main mitochondrial diseases with cardiac involvement.

Syndrome	Causative Genes	Inheritance Pattern	Clinical Manifestations	Onset
Leigh syndrome	More than 80 genes in mitochondrial (MtDNA) and nuclear DNA (nDNA), including SURF1	AR (mainly)	Seizures, encephalopathy, failure to thrive, dysphagia, cardiac involvement (HCM or DCM; valvular disease, arrhythmia, conduction defect)	Childhood
Sengers syndrome	Acylglycerol kinase AGK (nDNA)	AR	Cataracts, HCM, skeletal myopathy, lactic acidosis	Childhood/adulthood
Kearns–Sayre syndrome (KSS)	MtDNA deletion	Maternal inheritance pattern	Neurological involvement (ataxia, dementia), diabetes mellitus, cardiac conduction disorders (possible onset with sudden death), pigmentary retinopathy	Adulthood
Mitochondrial myopathy, encephalopathy, lactic acidosis and stroke-like episodes (MELAS) syndrome	MtDNA mutations (m.3243A>Gin MT-TL1, and other pathogenetic variants in MT-TL1) or MT-TV and MT-TQ	Maternal inheritance pattern	Ataxia, seizures, stroke-like episodes, myopathy, lactis acidosis, HCM, LV noncompactation, pre-excitation, atrioventricular block deafness.	Adulthood
Leber hereditary optic neuropathy (LHON)	Mutations in Mt-DNA m.11778G>A (MT-ND4), m.14484T>C (MT-ND6) and m.3460G>A (MT-ND1)	AR	Visual loss, cardiac involvement (pre-excitation)	Adulthood

AR, autosomal recessive; DCM, dilated cardiomyopathy; HCM, hypertrophic cardiomyopathy; MtDNA, mitochondrial deoxyribonucleic Acid; nDNA, nuclear deoxyribonucleic acid.

**Table 3:** Therapeutic strategies (drugs) and their respective main mechanism of influence on mitochondrial function and AF.

Medication	Main Effect on Mithocondrial Function and AF
SGLT-2 inhibitors [22,23,24,25,26,27]	↓ ROS production; restoration of mitochondrial membrane potential; ↑ mitochondrial biogenesis acting on PGC-1, NRF-1, Mfn-1, and AMPK; regulation of intracellulare electrolyte balance; ↓ in myocardial remodeling and fibrosis acting on TGF-beta/smad and NRF2/ARE; ↓ AF inducibility and in AF incidence
GLP1R antagonists [27,28,29,30,31]	↓ ROS production and ↑ in ROS scavengers’ mechanisms; antiapoptotic effects acting on cAMP/Epac/PI3K/Akt pathway; ↓ in myocardial remodeling and fibrosis; ↓ AF inducibility in animal models, contrasting data on humans
DDP-4 inhibitors [32,33,34,35,36,37,38]	↓ mitochondrial ROS production; ↓ mitochondrial membrane depolarization; ↑ mitochondrial biogenesis acting on PGC-1 /NRF1/Tfam; ↓ AF inducibility in animal models, contrasting data on humans
Metformin [39,40]	↑ mitochondrial oxygen consumption and activity of complexes I, II, and IV; ↓ atrial remodeling by activating the AMPK/PGC-1/PPAR; ↓ of AF incidence by 19%
Thiazolidinediones [41,42,43,44]	↓ oxidative stress; ↓ mitochondrial apoptotic signaling acting on PPAR; ↓ atrial remodeling; ↑ ion channel function (ICa and INa); ↓ AF inducibility in animal models, contrasting data on humans
Statins [45,46,47]	↓ oxidative stress through ↓ Rho/ROCK pathways, ↑ PI(3)K/Akt pathway, and ↓ NAD(P)H oxidase activity; ↓incidence of AF by 19%
Fibrates [48]	↑ mitochondrial function acting on PPAR/PGC-1; ↓ atrial remodeling and inducibility of AF prolonging atrial refractory period
Omega 3 fatty acids [48]	↓ ROS production; regulation of ion channels and cardiac electrical activity
Trimetazidine [49,50]	↓ miotochondrial ROS production by activate complex I and ETC; ↑ in mitochondrial biogenesis acting on PPAR/PGC-1α; improvement on mitochondrial fusion/fission dynamics acting on Mfn-1/Drp1/Opa-1; ↓ atrial remodeling; ↓ AF inducibility and duration in ischemic conditions
Ranolazine [51]	↓ mitochondrial ROS production due to inhibition of fatty acid oxidation; antiarrhythmic proprieties due to action on sodium and potassium channels
Carvedilol [48]	Block on alfa1 and beta1 adrenergic receptors; antioxidative proprieties
ACE-I, ARB, and AT1R blocker [48]	↓ROS production by XO and NADPH oxidase, induced by AngII; atabilization of cellular electrical proprieties blocking of the NF-κB action on SCN5A
Febuxostat and Allopurinol [52,53]	↓of oxidative stress inhibiting XO; ↓of AF susceptibility inhibiting ox-Ca^2+^-calmodulin-dependent protein-kinase type-II (CaMKII)
Ubiquinone (CoQ10) [54,55]	Cofactor is involved in electron transport within the respiratory chain; anti-inflammatory and anti-oxidant activity.

ACE-I: angiotensin converter enzyme inhibitors; AF: atrial fibrillation; AMPK: adenosine monophosphate kinase; AngII: angiotensin II; ARB: angiotensin receptors blockers; AT1R: angiotensin II receptor 1; cAMP: cyclic adenosine monophosphate; CoQ10: coenzyme Q 10; DPP-4i: dipeptidyl peptidase-4 inhibitors; Drp: dystrophin-Related Protein; Epac: Rap guanine nucleotide exchange factor 3; GLP1RA: glucagon-like peptide receptor antagonists; Mfn: mitofusin; NADPH: nicotinamide adenine dinucleotide phosphate; NF-kB: nuclear transcription factor-B; NRF: nuclear respiratory factor; Opa1: mitochondrial dynamin like GTPase; PGC: peroxisome proliferator-activated receptor-gamma coactivator; PI3K: phosphoinositide 3-kinase; PPAR: peroxisome proliferator-activated receptors; ROS: reactive oxygen species; Rho/ROCK: rhodopsin/Rho-associated protein kinase; SCN5A: sodium channel protein type 5 subunit alpha; SGLT-2: sodium/glucose cotransporter 2; TGF-beta: transforming growth factor-beta; Tfam: transcription factor A mitochondrial; akt: serine/threonine kinase 1; XO: xanthine oxidase; ↑: increase; ↓: reduction.

**Table 4:** Therapeutic strategies (nutraceuticals) and their respective main mechanism of influence on mitochondrial function and AF.

Medication	Main Effect on Mithocondrial Function and AF
Ubiquinone (CoQ10) [54,55]	Cofactor involved in electron transport within the respiratory chain; anti-inflammatory and anti-oxidant activity
Vitamin C and E [65]	Anti-inflammatory and anti-oxidant activity; ↓ post-surgical AF and AF recurrence after electrical cardioversion
N-acetyl cysteine [65]	↓ risk of AF by ↑ the density of L-type calcium current
L-glutamine [66]	↓ ROS production and stabilize the microtubule network through heightened heat-shock protein (HSP) expression.
Costunolide [48]	↑ mitochondrial function and ↓ in ROS production; anti-inflammatory and anti-fibrotic properties
Andrographolide [48]	↑ mitochondrial function and ↓ in ROS production; anti-inflammatory proprieties through regulation of calcium homeostasis genes

AF: atrial fibrillation; ROS: reactive oxidative species; ↑: increase; ↓: reduction.

**Table 5:** Antioxidant proprieties and effects on mitochondrial function of direct oral anticoagulation (DOAC).

Medication	Mechanism	Effect on Mitochondrial Function
Rivaroxaban [5,81,84,87,88,89,99]	Factor Xa inhibitor	↓ ROS production; restoration of mitochondrial membrane potential; ↑ mitophagy; ↑ citrate synthase; ↑ cytochrome C oxidase
Edoxaban [77,90,101]	Factor Xa inhibitor	↓ ROS production; ↑ mitochondrial oxigen consumption; ↑ ATP production; ↓ atrial remodeling
Apixaban [92,102]	Factor Xa inhibitor	↓ ROS production
Dabigatran [100]	Thrombin inhibitor	↓ ROS production; ↓ ROS-induced DNA strand breakage; ↓ SOD

ATP: adenosine triphosphate; DNA: deoxyribonucleic acid; ROS: reactive oxygen species; SOD: superoxide dismutase; ↑: increase; ↑: reduction.
